# Correlation analysis of carotid artery intima-media thickness, serum 25(OH)D and men with erectile dysfunction

**DOI:** 10.3389/fendo.2022.1027430

**Published:** 2022-10-06

**Authors:** Jun-hao Zhang, Wei Li, Cheng-yue Wang, An-ni Zhang, Ben-zhong Jia, Ya-wei Li, Zhen-duo Shi, Kai-fa Tang, Cong-hui Han

**Affiliations:** ^1^ Suzhou Medical College of Soochow University, Suzhou, China; ^2^ Department of Urology, Affiliated Hospital of Guizhou Medical University, Guiyang, China; ^3^ Department of Neurology, Affiliated Hospital of Guizhou Medical University, Guiyang, China; ^4^ Department of Oncology, the Affiliated Cancer Hospital of Guizhou Medical University, Guiyang, China; ^5^ Department of Urology, Xuzhou Central Hospital, Xuzhou, China

**Keywords:** vitamin D, erectile dysfunction, carotid artery intima-media thickness, CIMT, 25(OH)D

## Abstract

Our goal is to investigate the connection between serum 25(OH)D and carotid artery intima-media thickness (CIMT) in men with erectile dysfunction (ED).Serum 25(OH)D and CIMT were measured in 124 participants with erectile dysfunction and 39 healthy controls. The relationship between them and different patient-related parameters and disease-related parameters was studied. Compared with the control group and mild ED group, the level of serum 25(OH)D in moderate ED group and severe ED group decreased significantly(*P*<0.05). The CIMT values of moderate ED group and severe ED group were higher than those of the control group(*P*<0.05). The CIMT value of severe ED group was significantly higher than that of mild ED group(*P*<0.05). IIEF-5 score was positively correlated with serum 25(OH)D level, but negatively correlated with CIMT value(*P*<0.05). After adjusting for the influence of confounding factors, The CIMT values, 25(OH)D and IIEF-5 score were substantially associated(*P*<0.05). The serum level of 25(OH)D and IIEF-5 score were positively correlated, while the CIMT values and IIEF-5 score were negatively correlated. The level of serum 25(OH)D should be analyzed in men with ED, especially in patients with vasculogenic ED, and supplementation is recommended for those who were with vitamin D deficiency.

## Introduction

Erectile dysfunction (ED), is defined as that inability to achieve or maintain an erection that is firm enough to engage in sexual activity. Impotence is another term that has been used occasionally but is now less common. Nowadays, ED has gradually become one of the important diseases perplexing men all over the world. In recent years, studies from the United States, Britain and Italy have all shown that the onset of ED is gradually getting younger (1). Physiological process of penile erection is a vascular response under neuromodulation. Generally, sexual stimulation signals are transmitted to penile tissue, which will cause nerve terminals and endothelial cells to release bioactive factors such as nitric oxide (NO), and induce spongy smooth muscle relaxation, congestion and swelling of spongy smooth muscle. At the same time, the erectile penis compresses the vein and prevents blood flowing back from the corpora cavernous (2). Finally, the penis reaches and maintains sufficient rigidity to facilitate sexual intercourse. Therefore, when there is an obstacle in the process of blood entering the penis, it can give rise to the erectile function.

Carotid intima-media thickness (CIMT), which is thought to be an accurate predictor of systemic atherosclerosis and an objective measure of early atherosclerosis, is intimately associated with the development of cardiovascular disease (3). Sibal have demonstrated the role of CIMT in predicting cardiovascular disease (CVD) (4). At present, studies have shown that vascular diseases such as coronary heart disease, atherosclerosis and other vascular lesions are closely related to ED (5). Yao found that ED may be the earliest clinical manifestation when vascular lesions occur (6). In other words, the severity of ED may be associated with the risk of CVD, which seems to suggest that the severity of ED may also be associated with CIMT.

Vitamin D has mostly been recognized for its function in controlling calcium homeostasis and bone metabolism as a type of fat-soluble steroid hormone. The predominant form of vitamin D found in human body is 25-hydroxyvitamin D(25(OH)D). Numerous studies have pointed out that low serum vitamin D level is closely related to cardiovascular system, erectile function, and endothelial function (7–9). Monteiro proposed that vitamin D deficiency was associated with atherosclerosis (10). The relevance of vitamin D deficiency (defined as serum 25(OH)D level< 20 ng/ml) and vitamin D insufficient (defined as serum 25(OH)D level< 25 ng/ml) to public health is indisputable, although there is still controversy about optimal vitamin D status. Maintenance of normal serum vitamin D levels is the constituting principal focus of public health strategies.

However, it is still unclear how serum 25(OH)D levels, CIMT values, and erectile function are related. In this study, we wanted to investigate the connections between the three.

## Materials and methods

### Patients

This study was conducted on 163 Chinese Han males who participated in physical examination in the Affiliated Hospital of Guizhou Medical University from October 2021 to January 2022. The average age of all subjects was 30-60years(45.41 ± 7.44). All participants denied the use of Phosphodiesterase type-5 inhibitor(PDE5i) and vitamin D supplements. And all of them were subjected to detailed history taking, including smoking history (smoking ≥ 1 cigarette/D, time ≥ 6 months) and drinking history (alcohol intake > 25g/D), clinical examination, measurement of systolic blood pressure (SBP) and diastolic blood pressure (DBP), and determination of Body mass index (BMI). All participants were divided into four groups according to the IIEF-5 score, namely control group (IIEF>21), mild ED group (12≤IIEF ≤ 21), moderate ED group (8≤IIEF ≤ 11), severe ED group (0<IIEF ≤ 7).

Exclusion criteria included structural deformities of the penis like hypospadias. Medications-inducing ED such as antidepressants, antihypertensive, antiandrogens, neurogenic and psychogenic illnesses as well as abnormal serum testosterone levels were considered exclusion criteria. Patients who were underweight, those with type II diabetes, and those with illnesses known to influence 25(OH)D levels were also eliminated. Participants with a history of coronary artery disease, hypertension, hypogonadism, renal failure, autoimmune or inflammatory illnesses, or infection during the last six months were excluded. Similarly, all patients denied lower urinary tract symptoms(LUTS), prostatitis and prostatic hyperplasia in the past six months.

### Laboratory measurements

5 ml of peripheral venous blood was taken from each subject on an empty stomach in the morning. Fasting blood glucose (FBG), triglyceride (TG), total cholesterol (TC),high density lipoprotein cholesterol (HDL-C),low density lipoprotein cholesterol (LDL-C) and other biochemical indicators,were uniformly detected by the chemical automatic analyzer of the laboratory of the hospital.Serum 25(OH)D concentration was detected by enzyme-linked immunoassay (ELISA) (purchased from Shanghai Xitang Biotechnology Co. Ltd.).

### Evaluation of erectile dysfunction

The international index of erectile function-5(IIEF-5) score was used to evaluate the erectile function of the subjects. If the IIEF-5 score ranged from 22 to 25, the subjects were considered to have no ED. When IIEF-5 score <21, different degrees of ED was diagnosed. The severity of ED was classified according to IIEF-5 score, namely severe (0-7 points), moderate (8-14 points) and mild (15-21 points) (11).

### Measurement of CIMT

CIMT was measured for all participants. CIMT examination was performed by doctor from ultrasound department in a supine position with the head slightly overstretched and rotated to the contralateral side. In accordance with the Vascular Ultrasound Guidelines, CIMT measurements were automatically obtained using an ultrasonic system with a 5~13 MHz linear phased array probes in B‐mode pulsed doppler mode and colour mode. It measures the distance between two echogenic lines separated by the echo gap of the distal segment wall of the common carotid artery. CIMT was expressed as the mean the measurement of intima-media thickness of the left and right carotid arteries. Be careful to keep the frequency level the same for each patient.

### Statistical analysis

Data were analyzed by SPSS 26.0 statistical software. Categorical variables were described in numbers and percentages and compared with chi-square test.Kolmogorov-Smirnov test was used to verify the normality of the distribution of quantitative variables. The quantitative data of normal distribution were described by mean ± standard deviation and compared by one-way ANOVA, whereas the quantitative data of abnormal distribution were described by median (quartile range) and compared by non-parametric Kruskal-Wallis test. Spearman correlation was used to analyze the influencing factors of IIEF-5 score. Multiple linear regression was used to eliminate the influence of confounding factors and independently analyze the influencing factors associated with IIEF-5 score. Results were considered significant at *P*<0.05.

## Results

### Clinical data and laboratory parameters

**Figure 1:**
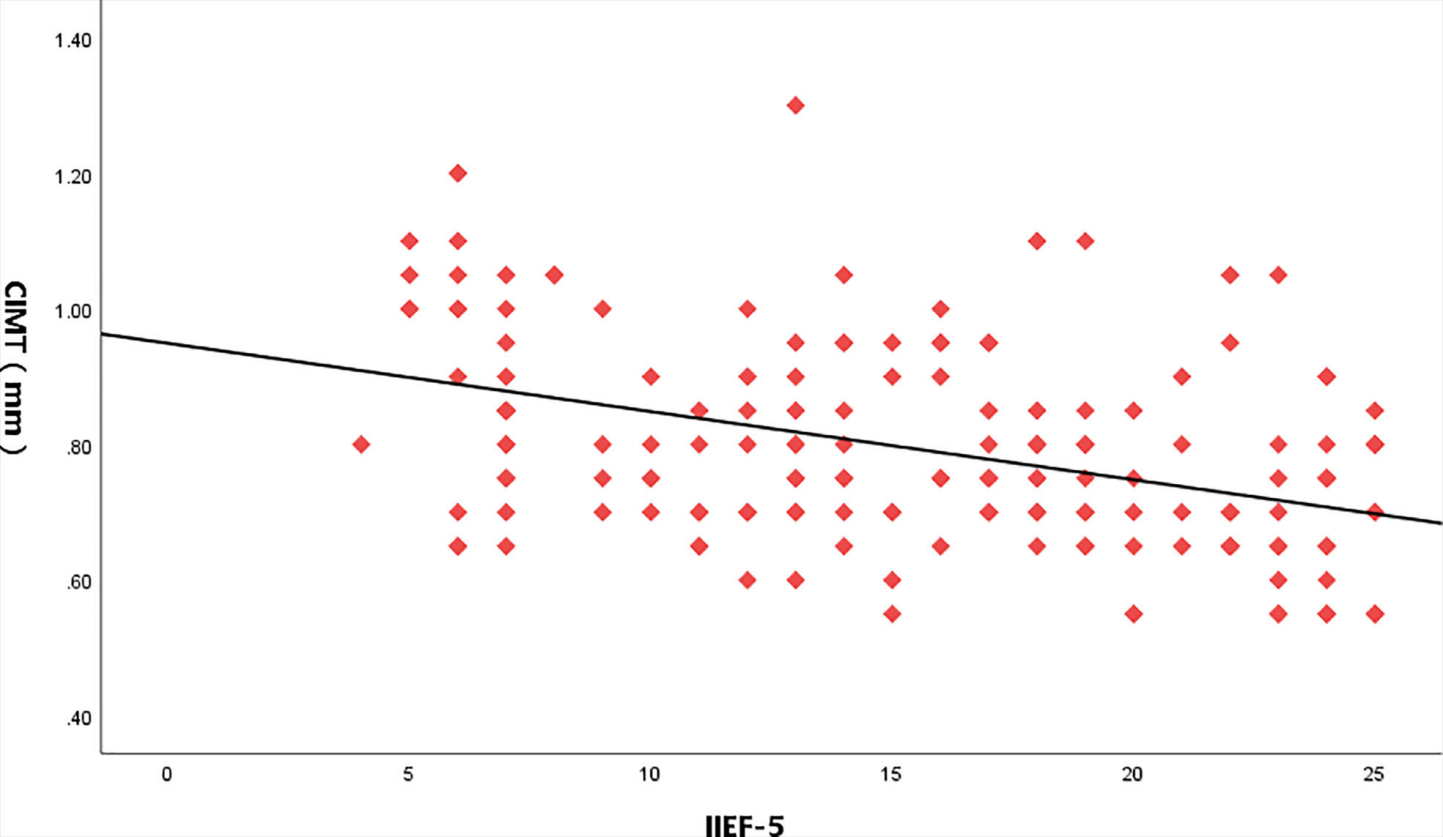
Serum 25(OH)D levels in healthy controls, mild ED, moderate ED, Severe ED. "#" is the comparison with the control group, P<0.05. "▲" is the comparison with the Mild ED group, P<0.05.

**Figure 2:**
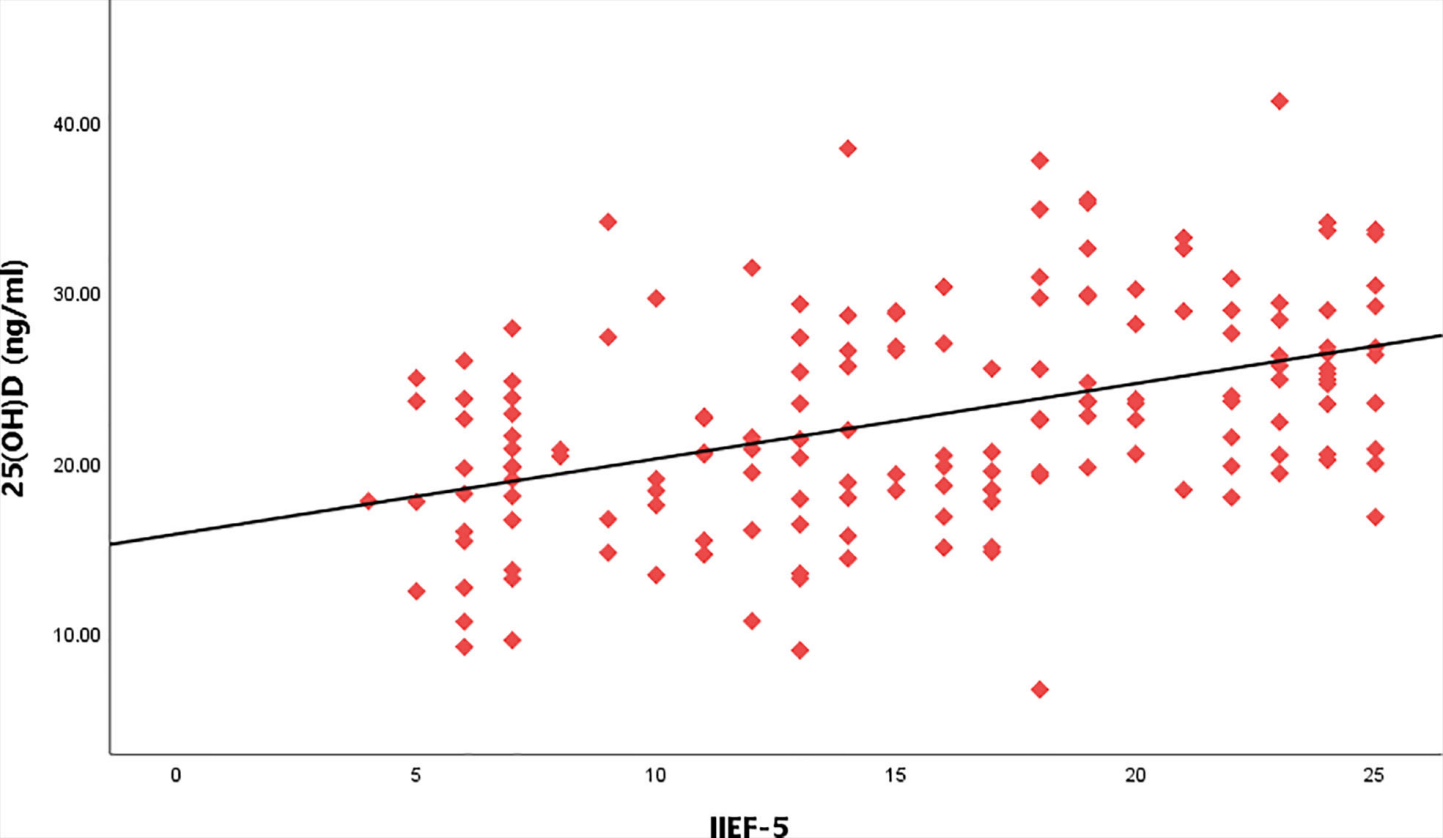
CIMT values in healthy controls, mild ED, moderate ED, Severe ED. "#" is the comparison with the control group, P<0.05. "▲" is the comparison with the Mild ED group, P<0.05.

According to the IIEF-5 score, 163 participants were divided into 4 groups, including 29 in the control group, 44 in the mild ED group, 51 in the moderate ED group, and 39 in the severe ED group.There is no significant difference in age, BMI, SBP, DBP, glucose, serum creatinine(Cr), Cholesterol, HDL, LDL, smoking and drinking among the four groups(*P>*0.05). The level of serum 25(OH)D in moderate and severe ED groups was lower than that in control group(*P*<0.05) (**Figure 1**). CIMT values in moderate and severe ED groups were significantly higher compared with control group(*P*<0.05) (**Figure 2**). Futhermore, CIMT value in severe ED group was significantly higher than that in mild ED group(*P*<0.05) (**Table 1**).

**Table 1:** Clinical and laboratory features of participants.

	Controls	Mild ED	Moderate ED	Severe ED
	(n=39)	(n=51)	(n=44)	(n=29)
Age	45.26 ± 6.96	45.78 ± 8.18	45.16 ± 6.74	45.34 ± 8.05
BMI	23.46 ± 2.88	23.62 ± 3.15	23.19 ± 2.27	23.81 ± 3.14
SBP(mmHg)	126.38 ± 12.39	127.12 ± 14.41	124.75 ± 11.36	128.83 ± 13.50
DBP(mmHg)	71.10 ± 7.50	71.92 ± 9.53	70.66 ± 6.44	72.07 ± 7.97
FBG(mmol/L)	4.96(4.66-5.12)	5.12(4.60-5.77)	5.19(4.47-5.67)	5.15(4.83-6.08)
Cr(umol/L)	74.56 ± 12.81	74.00 ± 11.65	75.23 ± 12.86	74.69 ± 10.30
UA(umol/L)	329.23 ± 59.57	320.43 ± 86.28	333.25 ± 71.66	317.52 ± 65.94
TG (mmol/L)	1.46(1.23-1.71)	1.58(1.29-1.89)	1.62(1.43-1.89)	1.68(1.26-2.55)
TC(mmol/L)	4.40 ± 0.58	4.37 ± 0.79	4.41 ± 0.72	4.39 ± 0.71
HDL-C(mmol/L)	1.19(1.04-1.32)	1.08(0.96-1.24)	1.16(0.99-1.27)	1.07(0.93-1.23)
LDL-C(mmol/L)	2.94(2.52-3.23)	2.97(2.39-3.33)	3.07(2.69-3.33)	3.05(2.66-3.56)
25(OH)D (ng/ml)	25.81 ± 5.18	24.18 ± 6.50	20.75 ± 6.27^ab^	18.64 ± 5.13^ab^
CIMT(mm)	0.70(0.60-0.80)	0.75(0.70-0.85)	0.80(0.70-0.90)^a^	0.90(0.78-1.03)^ab^
Smoking(%)	15(38.5%)	19(37.3%)	17(38.6%)	11(37.9%)
Drinking(%)	10(25.6%)	14(27.5%)	12(27.3%)	7(24.1%)

“a” is the comparison with the contorl group,P<0.05; “b” is the comparison with the mild ED group,P<0.05.

### Correlation analysis between IIEF-5 score and different indicators

As a result of Spearman correlation analysis, IIEF-5 score was positively correlated with serum 25(OH)D levels, but negatively correlated with CIMT values and triglyceride(*P<*0.05) (**Table 2**).

**Table 2:** Correlation analysis between IIEF-5 score and different indicators.

Variable	R	P
CIMT(mm)	-0.412	0.000
25(OH)D (ng/ml)	0.430	0.000
Age	0.019	0.814
BMI	-0.009	0.907
SBP(mmHg)	-0.015	0.846
DBP(mmHg)	0.010	0.899
FBG(mmol/L)	-0.080	0.310
Cr(umol/L)	0.005	0.951
UA(umol/L)	0.007	0.927
TG(mmol/L)	-.0199	0.011
TC(mmol/L)	0.010	0.897
HDL-C(mmol/L)	0.134	0.089
LDL-C(mmol/L)	-0.093	0.236

### Correlation analysis between IIEF-5 score, serum 25(OH)D level and CIMT values

**Figure 3:**
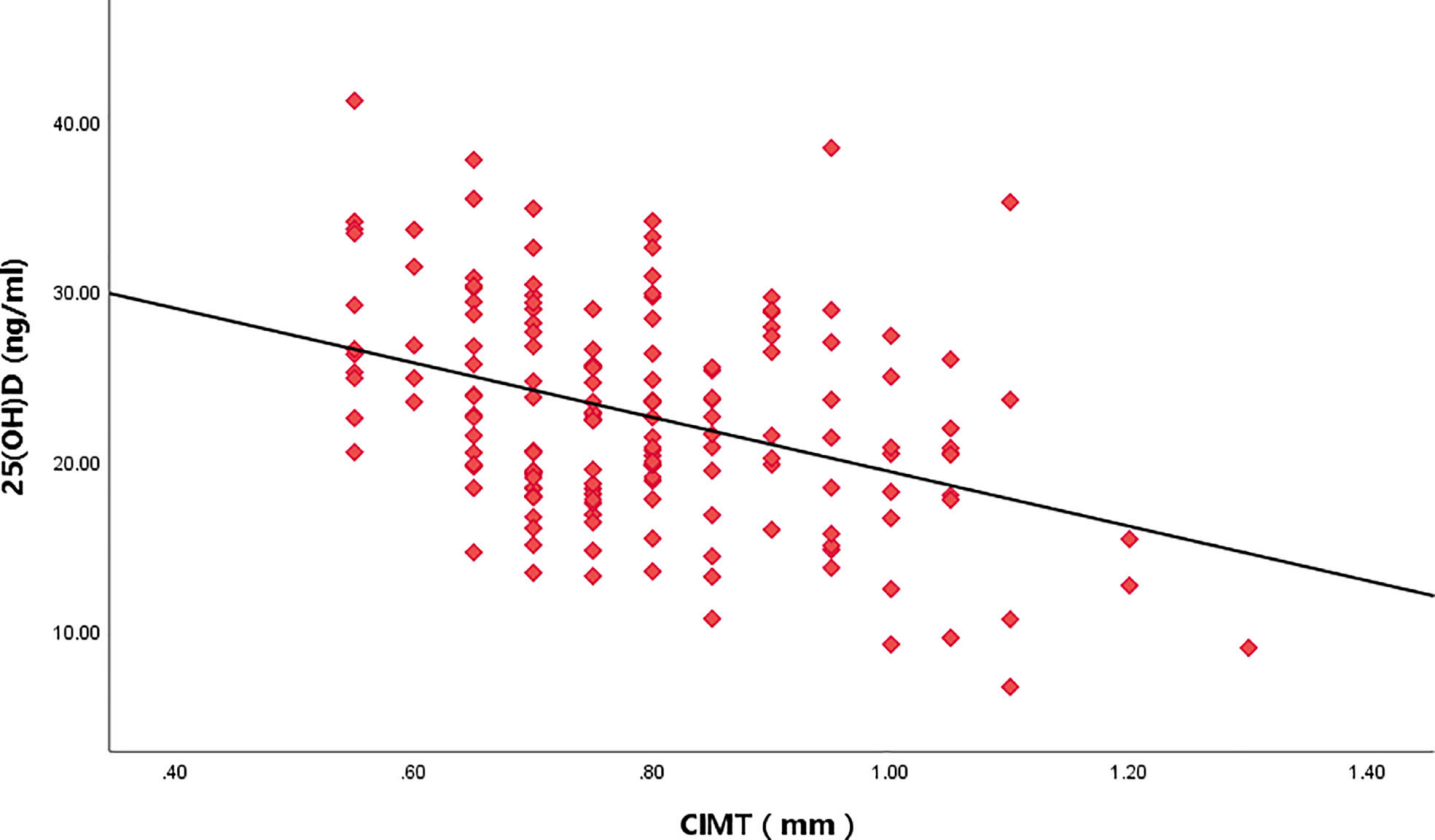
Relationship between IIEF-5score and CIMT values.

**Figure 4:**
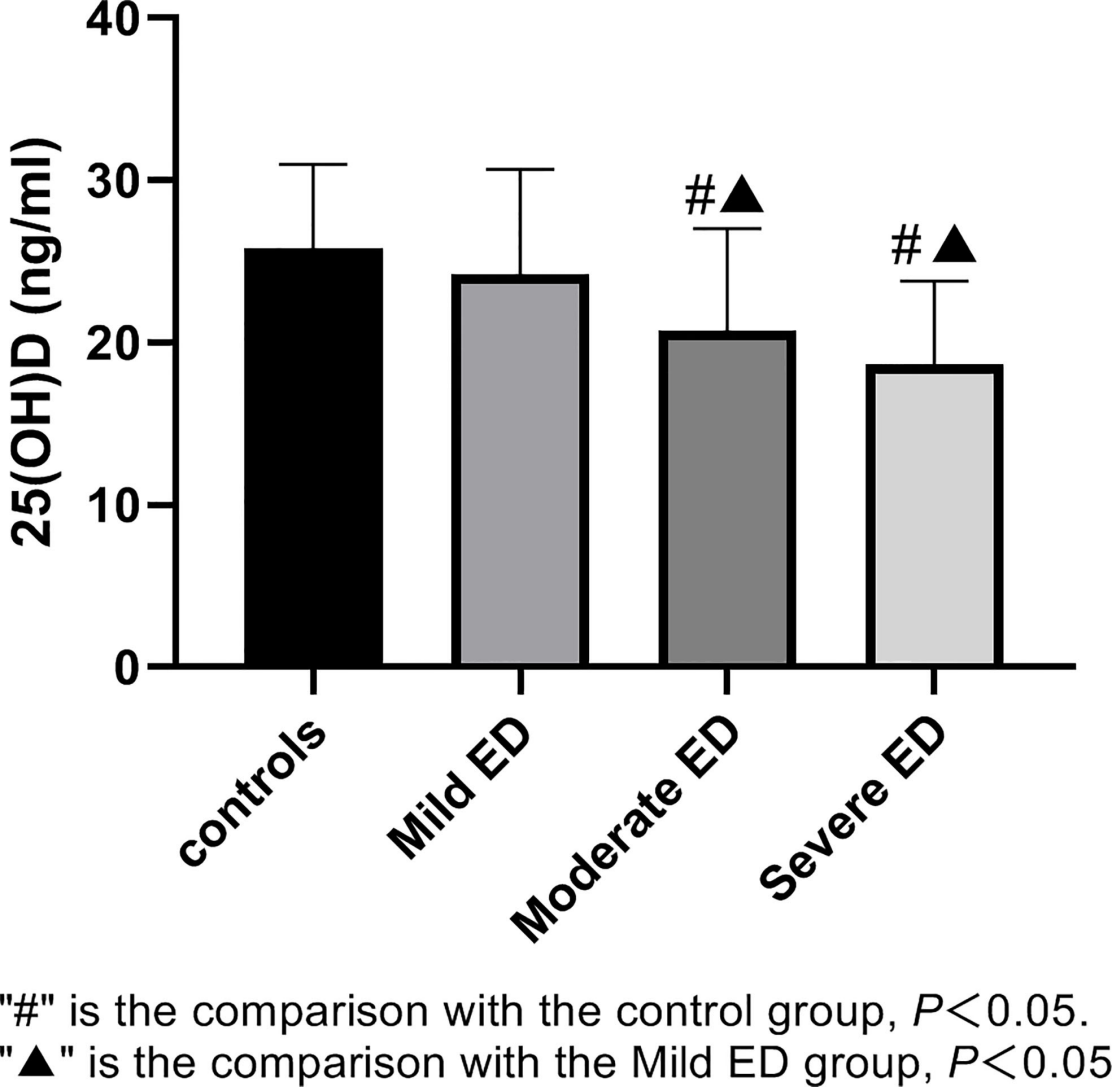
Relationship between IIEF-5score and serum 25(OH)D level.

**Figure 5:**
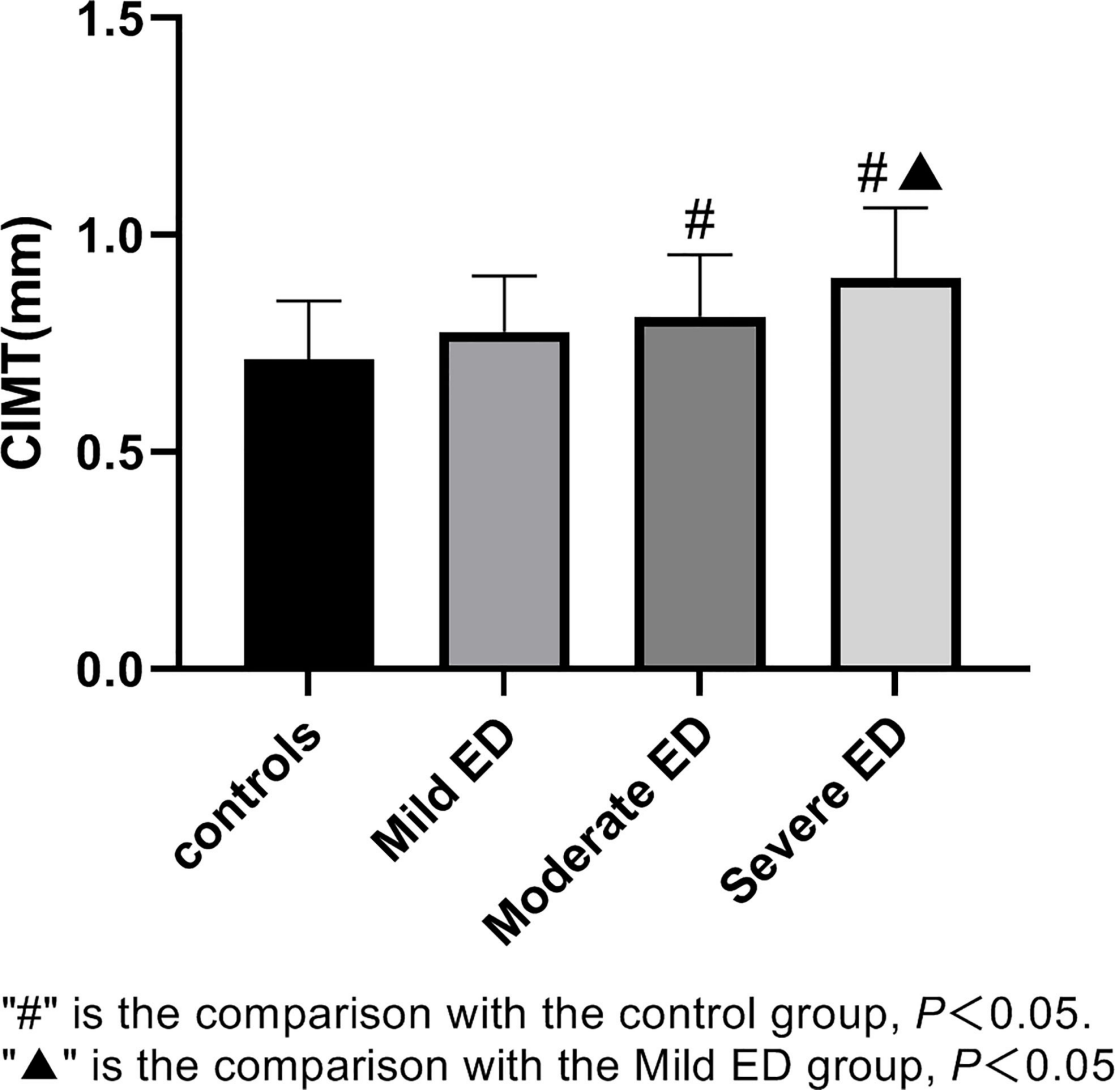
Relationship between serum 25(OH)D level and CIMT.

Taking the IIEF-5 score as the dependent variable and 25(OH)D, CIMT, and triglyceride as the independent variables, the results of multiple linear regression analysis showed that after adjusting for the confounding factors of triglyceride, IIEF-5 score was significantly correlated with the CIMT and serum 25(OH)D(*P*<0.05) (**Table 3** and **Figures 3**–**5**).

**Table 3:** Correlation analysis between IIEF-5 score,serum 25(OH)D level and CIMT values.

Variable	B	Std.Error	β	t	95% CI	P
(constant)	17.983	3.387	–	3.776	0.558~1.418	0.000
25(OH)D(ng/ml)	0.309	0.071	0.319	4.325	0.168~0.450	0.000
CIMT(mm)	-11.889	3.018	-0.290	-3.939	-17.85~-5.928	0.000

## Discussion

The incidence of ED increases with age. Although ED is not life-threatening, it can negatively impact the quality of life of the patients and their partners, especially in young men. Erectile function relays on complex interplay of vessels and nerves. The cavernosal branches of the internal pudendal artery supply the majority of the blood to the penis, while a network of tiny, easily compressed venules is responsible for venous outflow. Parasympathetic activity from the spinal cord’s sacral segments causes a cascade of processes to start when arousal happens, releasing nitric oxide and raising the level of cyclic guanosine monophosphate(cGMP) inside the cell. Increased cGMP results in relaxation of vascular smooth muscle and increases blood into the corpora cavernosa. Increased pressure in the corpus cavernosum results from the rapid entry of blood compressing the venule network and decreasing venous outflow, eventually resulting in an erection. Therefore, any condition that harms the neuronal or vascular circuits that support erections can lead to erectile dysfunction.

### Vitamin D and erectile dysfunction

According to the World Health Organization (WHO), when 25(OH)D concentrations are above 25ng/ml, it is considered normal. Vitamin D deficiency is defined as 25(OH)D concentrations below 20 ng/ml and vitamin D insufficiency as 25(OH)D concentrations between 20 and 25 ng/ml. Vitamin D deficiency to public health is indisputable. In this study, we discovered that participants with moderate and severe ED had lower levels of 25(OH)D, respectively, while 25(OH)D level was basically normal in participants with mild ED, implying that vitamin D deficiency may exist in ED patients, especially in patients with severe ED. Moreover, the 25(OH)D level and IIEF-5 score had a positive connection. Vitamin D deficiency and vitamin D insufficiency were currently considered to exist worldwide (12). Both Caretta’s (13) and Farag’s (14) studies found that the serum 25(OH)D level in ED patients was relatively low. According to Culha (15), there is a positive correlation between 25(OH)D levels and erectile function, which is consistent with our findings. However, in a meta-analysis (16), it was found that 25(OH)D levels did not show any significant difference between patients with and without ED. Similarly, the relationship between vitamin D levels and ED risk was not strongly confirmed in another meta-analysis (17). These conflicting results suggest that further well-designed studies with larger sample size included and outcome measures are needed in the future. Kim (8) reported that vitamin D was closely related to endothelial cell function, and mainly acted on endothelial cells in two main ways. First, vitamin D may control nitric oxide synthase(eNOS) expression and activity in endothelial cells, which could have an impact on how much NO is produced and released by endothelial cells; Second, vitamin D may mitigate damage of oxidative stress to endothelial cell function by regulating the antioxidant capacity of endothelial cells. The hypothesis of systemic endothelial dysfunction in patients with ED has been tested in several human studies, among which ED is the first clinical manifestation (17). Therefore, we speculated that vitamin D may affect the relaxation of vascular smooth muscle in cavernous tissue by regulating endothelial function, thereby affecting the erectile function eventually.

### Carotid artery intima-media thickness and erectile dysfunction

Endothelial and vascular smooth muscle cell dysfunction in the cavernous tissue is two of the primary causes of ED. When the function of vascular endothelial cells is impaired, the release of NO from endothelial cells is reduced (8), resulting in smooth muscle relaxation disorder and erectile dysfunction. At the same time, after the destruction of vascular endothelium, the lipid deposition between vascular walls increases, and after a series of reactions such as the impaired plasma coagulation and fibrinolysis mechanism, the increasing of growth factors, chemokines and so on, eventually leading to atherosclerosis (18). Thickening of CIMT, a well-known marker of atherosclerosis, occurs almost exclusively in arterial vessels (19), it is also an early change in atherosclerosis. Our study found that CIMT values in patients with ED were higher than those in control group, and we speculated that men with thickened CIMT may be at greater risk for ED. Mulla (20) reported in his study that CIMT was negatively correlated with IIEF-5 score, in other words, the thickness of CIMT in patients with ED was significantly thicker than that of healthy men, which was consistent with the results of our study. We suspected that the vessels in penile cavernous tissue of ED patients may also have lesions along with the vascular system of the whole body. Furthermore, CIMT can be used to predict the severity of ED and how well patients with vasculogenic ED respond to phosphodiesterases, according to additional research that has supported these findings (21). According to the findings, patients with elevated CIMT responded to tadalafil less favorably than those with normal CIMT. Tadalafil’s poor efficacy in patients with elevated CIMT (>0.67 mm) is most likely caused by endothelial dysfunction and structural abnormalities in artery walls that block the nitrite oxide route. Therefore, in ED patients with thickened CIMT, physicians should be warned that they may not respond well to phosphodiesterase 5 inhibitor(PDE5-I), and other treatment options such as penile sponge injection should be considered in these patients.

### Vitamin D and carotid artery intima-media thickness

In this study, comparing the moderate and severe ED groups to the control and mild ED groups, we discovered statistically significant drops in serum 25(OH)D levels, and we discovered a notably greater level of CIMT in the groups with moderate and severe ED. Serum 25(OH)D levels appeared to be inversely linked with CIMT. Similarly, Van (22) demonstrated that increased CIMT and low vitamin D levels were related. In 2017, A meta-analysis of 21 studies (23) revealed that patients with vitamin D insufficiency also had significantly greater CIMT and carotid plaque prevalence. A meta-analysis of an additional 11 trials (18) revealed that serum vitamin D levels were a preventative measure against carotid plaque. Uncertainty exists regarding the processes underlying the link between serum vitamin D and CIMT. However, researches (24, 25) has shown that a lack of vitamin D increases the risk of cardiovascular disease and stiffens the arteries. This might be because vitamin D helps to activate the renin-angiotensin system.

Combined with these results, we speculated that there may be a potential correlation between serum vitamin D level, CIMT and erectile function. These results of this study preliminarily verified our conjectures that erectile function was positively correlated with serum 25(OH)D level, and negatively correlated with CIMT, while serum 25(OH)D level was negatively correlated with CIMT. At this point, we wondered if vitamin D might cause vascular disease and ED. In Kim’s research, he reported that endothelial cells played a key role in regulating vascular homeostasis and hemodynamics, especially in vasodilation. Endothelial cells cause vascular smooth muscle relaxation mainly through production of eNOS and release of NO. The mechanism of penile erection happens to be the release of NO by non-adrenergic noncholinergic (NANC) nerve fibres. Following signaling pathways result in elevated cGMP levels, decreased intracellular Ca2+ levels, and relaxation of the smooth muscle cell (26, 27). Men finally have an erection as a result of the smooth muscle cells relaxing allowing blood to enter the lacunar gaps in the corpora cavernosa and compressing the unilateral inferior veins and restricting the vein’s outflow. Therefore, vitamin D may affect erectile function through its interaction with endothelial cells of corpora cavernosa, which will guide the direction of our further research.

It is worth noting that men with a reduced IIEF-5 score alone cannot be diagnosed as vasculogenic ED, it is equally important that psychogenic ED and endocrine ED should be excluded. Most of the participants in this study were men who came to hospital for routine physical examinations. Because of various reasons, the majority of participants refused further examinations to exclude organic ED, which is also the deficiency of this study and will be improved in the follow-up studies of our research group. Secondly, the blood collection period in this study was autumn and winter, so, factors, such as sunshine, season, climate, region may affect the fluctuation of serum 25(OH)D levels, which is planned to be further improved in the subsequent series of studies.

## Conclusion

In conclusion, this study found that erectile function was positively correlated with serum level of vitamin D, and negatively correlated with CIMT. The mechanism of vitamin D influencing on erectile function was still unknown, and whether CIMT can predict severity of erectile function, all these questions need to be further confirmed. Lower level of vitamin D may increase the risk of morbidity of ED by regulating endothelial function. Serum vitamin D level should be analyzed in men with ED, especially in patients with vasculogenic ED, and supplementation is recommended for those who were with vitamin D deficiency.

## Data availability statement

The original contributions presented in the study are included in the article/supplementary material. Further inquiries can be directed to the corresponding authors.

## Ethics statement

The studies involving human participants were reviewed and approved by The Ethics Committee of the Affiliated Hospital of Guizhou Medical University.

## Author contributions

J-HZ came up with the idea for the study, helped with its planning and data gathering, carried out the statistical analysis, and wrote the manuscript. The project was created by C-HH and K-FT, who also contributed with its planning, design, statistical analysis, and manuscript writing. Techniques were offered as support by C-YW and Z-DS. WL, A-NZ, B-ZJ, and Y-WL assisted gather the data and with the statistical analysis. All authors contributed to the article and approved the submitted version.

## Funding

This study was funded by the Medical Science and Technology Research Project of Guizhou Province provided by the Health Commission of Guizhou Province (NO.gzwkj2023-372) and the Medical Innovation team of Xuzhou city(No.XWCX201603).

## Conflict of interest

The authors declare that the research was conducted in the absence of any commercial or financial relationships that could be construed as a potential conflict of interest.

## Publisher’s note

All claims expressed in this article are solely those of the authors and do not necessarily represent those of their affiliated organizations, or those of the publisher, the editors and the reviewers. Any product that may be evaluated in this article, or claim that may be made by its manufacturer, is not guaranteed or endorsed by the publisher.
